# Predicting Hip Fracture Type With Cortical Bone Mapping (CBM) in the Osteoporotic Fractures in Men (MrOS) Study

**DOI:** 10.1002/jbmr.2552

**Published:** 2015-07-14

**Authors:** Graham M Treece, Andrew H Gee, Carol Tonkin, Susan K Ewing, Peggy M Cawthon, Dennis M Black, Kenneth ES Poole, ; for the Osteoporotic Fractures in Men (MrOS) Study

**Affiliations:** ^1^ Department of EngineeringUniversity of CambridgeCambridgeUK; ^2^ Research Scientist and Radiography ConsultantGranville FerryNova ScotiaCanada; ^3^ Department of Epidemiology and BiostatisticsUniversity of California, San FranciscoSan FranciscoCAUSA; ^4^ California Pacific Medical Center Research InstituteSan FranciscoCAUSA; ^5^ Department of MedicineUniversity of CambridgeCambridgeUK

**Keywords:** OSTEOPOROSIS, CORTICAL BONE MAPPING, QCT, FRACTURE RISK

## Abstract

Hip fracture risk is known to be related to material properties of the proximal femur, but fracture prediction studies adding richer quantitative computed tomography (QCT) measures to dual‐energy X‐ray (DXA)‐based methods have shown limited improvement. Fracture types have distinct relationships to predictors, but few studies have subdivided fracture into types, because this necessitates regional measurements and more fracture cases. This work makes use of cortical bone mapping (CBM) to accurately assess, with no prior anatomical presumptions, the distribution of properties related to fracture type. CBM uses QCT data to measure the cortical and trabecular properties, accurate even for thin cortices below the imaging resolution. The Osteoporotic Fractures in Men (MrOS) study is a predictive case‐cohort study of men over 65 years old: we analyze 99 fracture cases (44 trochanteric and 55 femoral neck) compared to a cohort of 308, randomly selected from 5994. To our knowledge, this is the largest QCT‐based predictive hip fracture study to date, and the first to incorporate CBM analysis into fracture prediction. We show that both cortical mass surface density and endocortical trabecular BMD are significantly different in fracture cases versus cohort, in regions appropriate to fracture type. We incorporate these regions into predictive models using Cox proportional hazards regression to estimate hazard ratios, and logistic regression to estimate area under the receiver operating characteristic curve (AUC). Adding CBM to DXA‐based BMD leads to a small but significant (*p* < 0.005) improvement in model prediction for any fracture, with AUC increasing from 0.78 to 0.79, assessed using leave‐one‐out cross‐validation. For specific fracture types, the improvement is more significant (*p* < 0.0001), with AUC increasing from 0.71 to 0.77 for trochanteric fractures and 0.76 to 0.82 for femoral neck fractures. In contrast, adding DXA‐based BMD to a CBM‐based predictive model does not result in any significant improvement. © 2015 The Authors. *Journal of Bone and Mineral Research* published by Wiley Periodicals, Inc. on behalf of the American Society for Bone and Mineral Research.

## Introduction

Hip fractures are the most common cause of acute orthopedic hospital admission in older people.^1^ Bone mineral density (BMD) is an important imaging marker that contributes to an individual's fracture risk, and is usually measured as areal BMD from dual‐energy X‐ray (DXA), though volumetric BMD from quantitative computed tomography (QCT) is a viable surrogate. Although BMD is specific^2^, ^3^ it lacks sensitivity,^(3–5)^ missing the majority who go on to fracture. There is now growing evidence that focal, structural weaknesses may predispose a hip to fracture.^(6–8)^ The distribution of both trabecular and cortical bone is critical in determining a femur's resistance to fracture.^(9–12)^ Drug treatment and exercise regimes targeted at reducing fracture risk result in changes that are focused in particular regions rather than dispersed over the whole bone.^13^, ^14^

Cortical bone mapping (CBM) is a technique that allows measurement of cortical variables from clinical QCT data; CBM has been presented and thoroughly validated in previous works.^15^, ^16^, ^17^ Using CBM, it is possible to accurately measure cortical thickness (CTh, mm), cortical mass surface density (CM, mg/cm^2^, the cortical mass per unit cortical surface area), cortical bone mineral density (CBMD, mg/cm^3^), and endocortical trabecular bone mineral density (ECTD, mg/cm^3^, the average density in the trabecular compartment close to the cortex). This is distinct from alternative measures in two important ways. First, it provides a genuinely local measurement of cortical properties: these are independently measured at many thousand locations distributed over the surface of the proximal femur. Particular cortical regions that may be related to particular types of fracture are found from a statistical analysis of these measurements, rather than pre‐grouping them into anatomical regions and testing each region in turn for significance. Such an analysis has been applied to hip fracture before,^18^ but using voxel‐based BMD rather than surface‐based cortical measurements. Second, measurements are accurate even when the cortex is much thinner than the extent of the CT imaging blur. In contrast, in most alternative techniques, for thin cortices (<3 mm) the measured “thickness” when based on thresholds (either locally variable or fixed), whether or not refined by morphological operators, is often closer to the width of the CT imaging blur, and the measured “density” (ie, the recorded cortical CT value) is in fact closer to cortical mass surface density, rather than true cortical BMD.

Here we apply CBM for fracture prediction, in a study of male subjects, some of whom have gone on to fracture their femur. QCT data from this study has been analyzed before using both DXA‐derived and QCT‐derived regional bone quantities,^19^ though with a different randomly selected cohort. This present work is the first application of CBM to a predictive fracture‐risk study. It also reports on a much larger number of cases than previously published results: 99 rather than the previously reported 40. As a result, we consider these cases in two separate fracture groups (trochanteric and neck fractures) for the first time in a male cohort, although there have been previous discriminatory analyses of fracture type in female cohorts.^11^, ^18^, ^20^ Using CBM we can show the different patterns of relationship between fracture type and cortical distributions and hence start to consider the assessment of not only whether a particular hip is at risk of fracture, but where that fracture is most likely to occur. This is of particular importance when considering that both exercise and drug regimes improve bone in specific areas: the eventual goal is to be able to match a particular individual at risk of fracture to the regime which will best ameliorate their specific fracture risks.

## Subjects and Methods

### Study design

The Osteoporotic Fractures in Men (MrOS) study^21^, ^22^ recruited 5994 men in the United States from March 2000 until April 2002. Eligible subjects from six clinical sites were 65 years or older, able to walk without assistance, and had not had bilateral hip replacement surgery. Various measurements at baseline included areal BMD at the hip by DXA (QDR 4500W; Hologic Inc., Bedford, MA, USA) and subject weight and height. Information about fractures was ascertained through questionnaires returned by the subjects every 4 months; however, any reported hip fractures were validated by centralized physician review of the radiology report or radiographs. Fracture type was classified as “femoral neck,” “subtrochanteric,” “intertrochanteric,” or “other.”

A subset of 3684 participants (61% of the MrOS cohort) also had QCT of the hip at baseline. This subset consisted of approximately the first 650 subjects at each of the six centers, in addition to all those from minority backgrounds. QCT scans were performed on a variety of machines, but according to a protocol delivering 512 × 512 pixel slices at 3 mm separation, covering the femoral head to 3.5 cm below the lesser trochanter. All scans included a calibration phantom (calcium hydroxyapatite at 150, 75, and 0 mg/cm^3^; Image Analysis Inc., Columbia, KY, USA) for converting from Hounsfield Units (HU) to BMD. A total of 3572 of these scans were successfully transferred to the study center for processing, and QCT‐derived volumetric BMD values were calculated from a further subset of 3358. Scanning and selection procedures, baseline statistics compared to the entire cohort, and details of the QCT‐derived measurements have been described fully elsewhere.^23^

The analysis of this study follows a case‐cohort design.^24^ The MrOS study team took the 3572 participants with hip QCT scans and removed any with previous hip fracture or hip replacement, leaving 3515. The cases were all 104 participants from this set with hip fractures as of February 2012. Three times this number (312) were taken as a random sample from the whole set to form the cohort, of which 10 had fractured and hence were also in the cases, leaving a total sample size of 406. Eight participants were excluded from the analysis because of problems with the QCT data (two incomplete scans, four severely misregistered scans, one incomplete phantom scan, and one extremely noisy reconstruction), leaving 398 in the final analysis. Of these 398, 308 were in the cohort and 99 were fracture cases. Fracture types were loosely grouped into two categories, giving 44 “trochanteric” (37 intertrochanteric, 4 subtrochanteric, and 3 other) and 55 “neck” fractures.

An additional set of 38 QCT scans was also supplied, for analyzing CBM reproducibility. This consisted of two independent scans at visits 3 months apart from each of 19 men.

### CBM

The first step in CBM is an approximate segmentation of each proximal femur from the QCT data for each subject. This segmentation is performed using in‐house–developed software Stradwin (free to download from http://mi.eng.cam.ac.uk/∼rwp/stradwin), and results in a triangulated surface mesh with between 5,000 and 15,000 vertices distributed uniformly over the proximal femoral surface. This is the only manual step in the CBM process. These vertices are used to establish the location and directions at which more accurate, fully automated, measurements of cortical variables (including more precise surface location) are made. Hence the manual segmentation is only required to be accurate to within 2 mm of the real femoral surface.

CBM measurements are then made at each vertex, again using in‐house Stradwin software. This is followed by non‐rigid registration of each femur from each subject to a canonical femur surface (derived from an average of many hundred femurs) and mapping of the CBM data to that surface, using another in‐house package wxRegSurf (free to download from http://mi.eng.cam.ac.uk/∼ahg/wxRegSurf). Statistical parametric mapping (SPM) is then applied, using the SurfStat^25^ package, to identify patches of differences in any of these variables associated with each fracture type (trochanteric or neck). Where both hips were available (in 358 out of 398 subjects) results were averaged over the left and right femur; otherwise, data was used from whichever hip was available. We have previously shown that (at least in older women), although there are some differences between left and right femurs from the same subject, these are relatively small and do not generally appear in areas associated with fracture.^26^

The general linear model variables used in SPM to discover patches for each of CTh, CM, CBMD, or ECTD, were group, fracture, age, weight, clinical site, and shape. Group was bimodal (case or cohort) whereas fracture was trimodal (none, trochanteric, neck). Clinical site was used to model any variations in scanner calibration and regional demographics. To model shape we used the five most significant shape modes: with size, these account for 86% of the total shape variance. However, we do not include size in the model because it is known to correlate with fracture, and would hence reduce the fracture‐sensitive CBM patches. The five shape modes are required as confounding variables to account for any systematic misregistration during the mapping process.^27^ For each cortical variable, we identified two patches (ie, the regions where there was significant contrast between the cohort and the specific fracture type): one patch for trochanteric fractures, and another for femoral neck fractures. Having identified these patches, the cortical data was averaged within the corresponding patch. This gives single values per patch, cortical variable, and subject. As a result of a prior discriminative study on a different data set,^11^, ^28^ it was decided in advance of this study only to use the CM and ECTD patches for analysis of hazard and odds ratios, giving four variables in all to carry forward into the next phase of statistical analysis. CTh and CBMD patches are nevertheless also reported for completeness.

**Figure 1:**
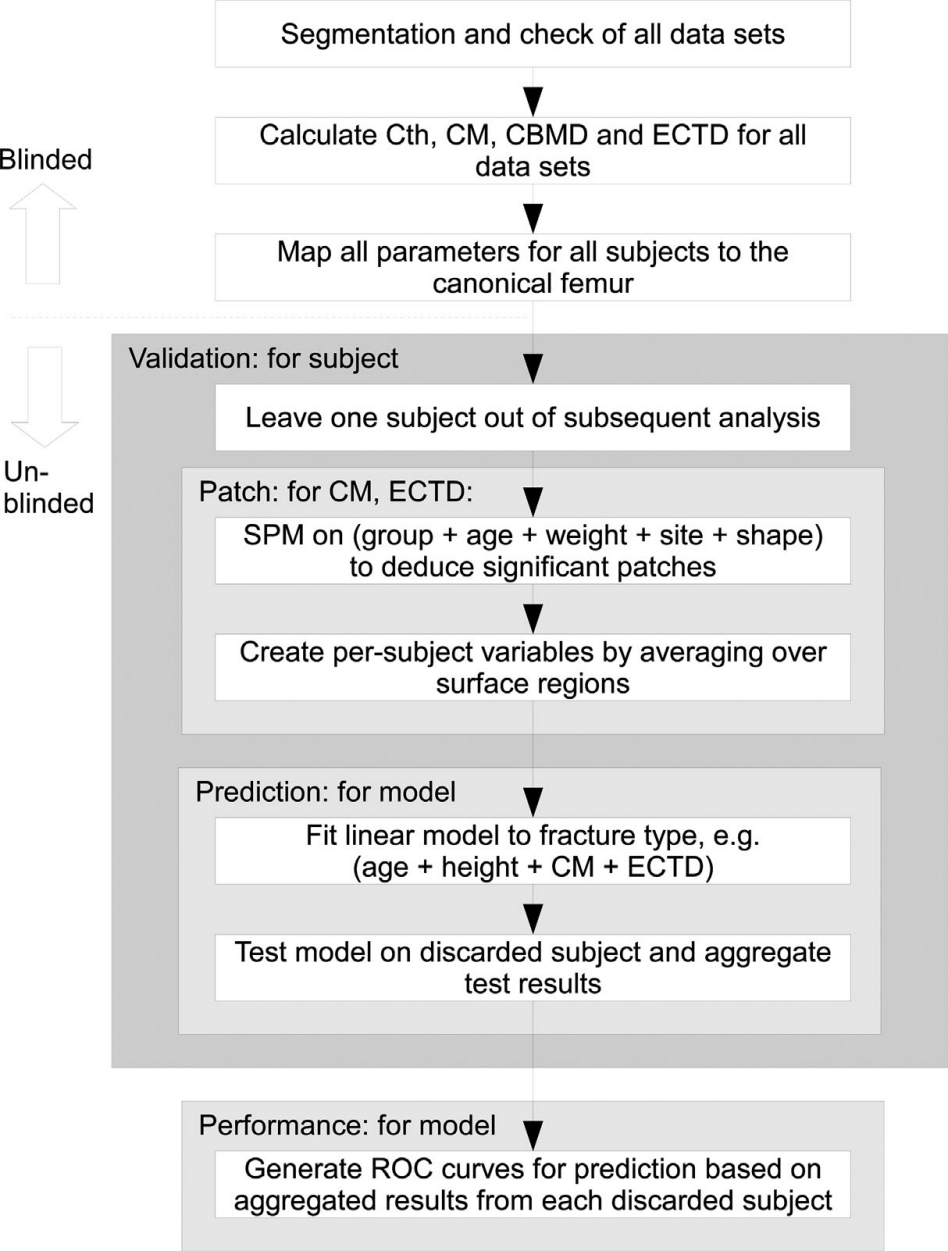
Flowchart describing the process for creating and validating predictive linear models based on CBM variables.

All stages previous to SPM analysis were carried out blinded to case and fracture status, and all the analysis (with the exception of some additional predictive models highlighted in the following section) was according to a study plan that had been previously submitted to, and approved by, the MrOS study group. The process is summarized in the top section of Fig. 1.

### Predictive models

Variables of interest were age, height, DXA‐based areal BMD (total hip [ThBMD] and femoral neck [FnBMD]), and QCT‐based integral volumetric BMD (total hip and femoral neck), as well as the CM and ECTD patches based on CBM analysis for trochanteric and neck fracture. Outcomes were either any fracture, or trochanteric fracture, or femoral neck fracture. QCT‐based BMD measurements were provided for 384 of the 398 subjects.

Modeling of the time to first incident hip fracture was performed using Cox proportional hazards regression with the Barlow weighting method and robust variance estimation, necessitated by the case‐cohort design. Hazard ratios were estimated for hip fracture for a 1 SD change in each imaging variable, adjusted for age, height, and clinical site. According to Barlow's method for weighting study participants in the pseudo‐likelihood, cases were weighted by 1 and subcohort controls were weighted by the inverse of the sampling fraction, where α = 308/3515 = 8.8%. Case‐cohort analyses for hazard ratios were conducted in SAS version 9.1 (SAS Institute, Inc., Cary, NC, USA).

In addition, we examined the ability of models involving different groups of variables to predict 10‐year fracture incidence, by performing either binomial (any fracture) or trichotomous multinomial (specific fracture type) logistic regression (MNR) for various models, allowing for age, height, and clinical site. Logistic models were created using MATLAB R2014a (The MathWorks, Inc., Natick, MA, USA). We calculated odds ratios for each variable individually. We investigated predictive models including no imaging variables, DXA‐derived BMD values only, QCT‐derived BMD values only, CBM patches only, DXA and CBM patches, and QCT and CBM patches. Receiver operating characteristic (ROC) curves were calculated with leave‐one‐out cross‐validation. Because the location of the CBM patches were also dependent on the data, SPM for calculating the patches was also included in the leave‐one‐out process. Hence, for each subject, fracture probability for each type of fracture was predicted from a model based on the remaining subjects, and also (where contained in the model) from CBM patches based on the remaining subjects. This process is summarized in the lower section of Fig. 1.

We used a significance level of *p* < 0.005 for hazard ratios, odds ratios, and for comparing models, as a conservative Bonferroni correction given that there are up to 10 variables under consideration in the main analysis (age, height, and all DXA, QCT, and CBM values).

The predictive models listed in this section were all determined prior to any data analysis as part of a plan submitted to the MrOS study group. This is particularly important for model selection, because overoptimistic results can be generated if selection of variables is based on model performance. Nevertheless, some additional models were selected after the analysis, to allow comparison with previous studies.^19^, ^29^ These models included other DXA‐based variables (trochanteric and intertrochanteric areal BMD, to give four in total) and QCT‐based variables (trochanteric, femoral neck, and total hip volumetric BMD in integral, cortical, and trabecular compartments, to give nine in total).

### Precision

Precision of the CBM variables was assessed with an additional set of QCT data that consisted of 38 scans of 19 subjects, with two independent scans of each subject. CBM variables were derived from these scans in the same way as for the other analyses above and mapped to the canonical femur model. Each scan was processed blinded to subject. The variance of the measurement error was assumed to be one‐half the variance of the measurement differences from each pair of scans of the same subject (because if the measurement error is normally distributed with zero mean, the difference between two samples of the same measurement has twice the variance of each of the samples alone). The measurement SD was calculated for each CBM variable, at each location on the canonical femur, because the precision was expected to vary with anatomical location. We also calculated the SD of the CBM variables after averaging these over the patches found in the SPM analysis.

## Results

Baseline data for no fracture and for the fracture cases is given in Table 1.

**Table 1:** Baseline Values for the Study

	No fracture			Fractures			
	(*n* = 299)^a^	All (*n* = 99)^a^		Trochanteric (*n* = 44)^a^		Neck (*n* = 55)^a^	
Quantity	Mean ± SD	Mean ± SD	*p*	Mean ± SD	*p*	Mean ± SD	*p*
Age (years)	73.4 ± 5.7	76.8 ± 5.8	<0.001	75.5 ± 5.7	0.025	77.8 ± 5.7	<0.001
Weight (kg)	84.6 ± 14.1	80.7 ± 13.0	0.017	78.8 ± 11.4	0.010	82.3 ± 14.1	0.263
Height (cm)	174.4 ± 7.3	174.3 ± 6.3	0.840	174.1 ± 6.0	0.758	174.4 ± 6.5	0.990
DXA ThBMD (g/cm^2^)	0.956 ± 0.132	0.837 ± 0.131	<0.001	0.827 ± 0.120	<0.001	0.844 ± 0.140	<0.001
DXA FnBMD (g/cm^2^)	0.786 ± 0.119	0.675 ± 0.105	<0.001	0.679 ± 0.107	<0.001	0.672 ± 0.104	<0.001
QCT ThBMD (g/cm^3^)	0.278 ± 0.049	0.236 ± 0.049	<0.001	0.233 ± 0.047	<0.001	0.238 ± 0.051	<0.001
QCT FnBMD (g/cm^3^)	0.286 ± 0.057	0.242 ± 0.053	<0.001	0.244 ± 0.052	<0.001	0.240 ± 0.053	<0.001

Significant differences of *p* are compared to no fracture.

^a^ QCT values were given for a subset of the data, with *n* = 288, 96, 43, and 53, respectively.

**Figure 2:**
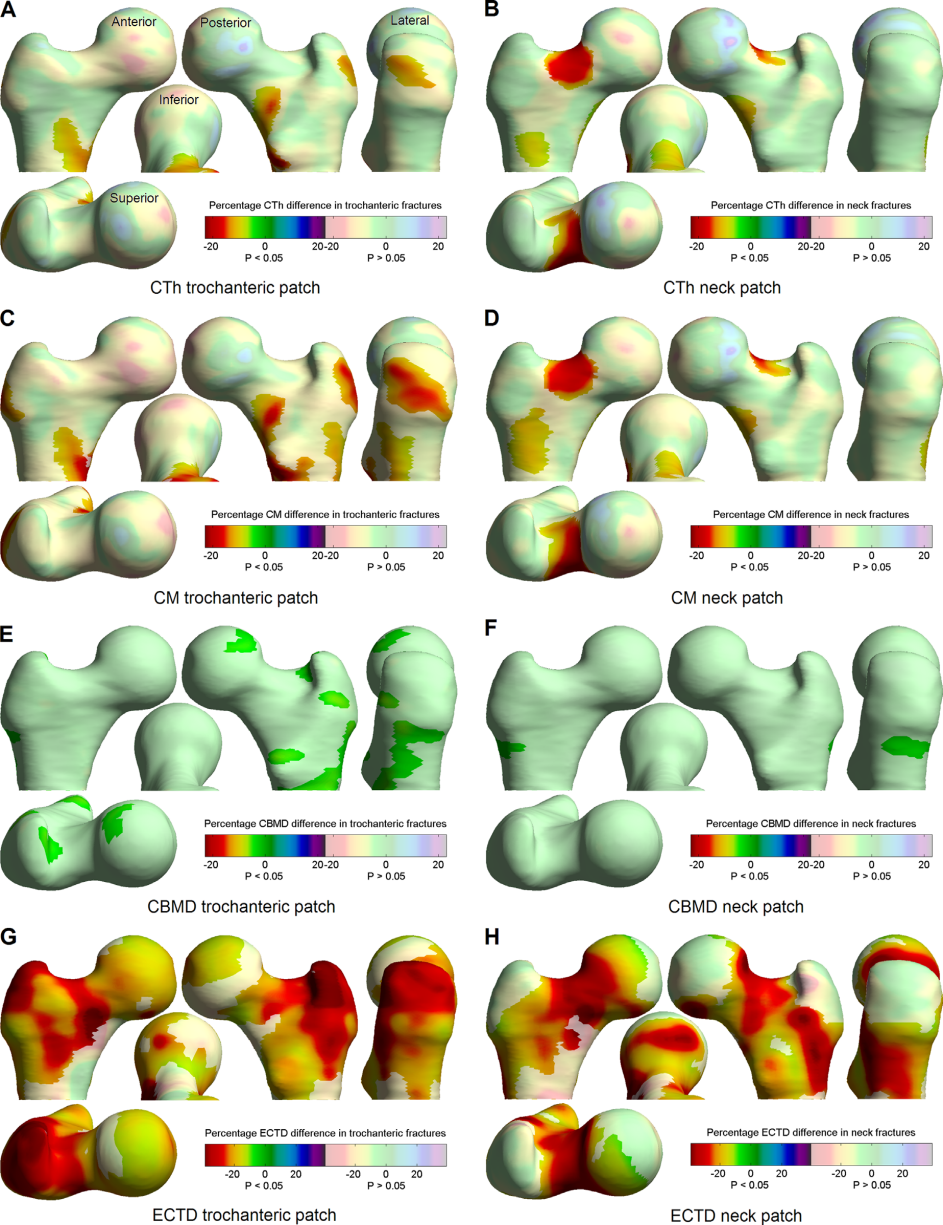
CBM effects related to each fracture type, shown as percentage differences between fracture cases and cohort. Paler colors indicate no significant relationship between fracture type and the CBM quantity. The left‐hand images concern trochanteric fracture, the right‐hand images concern neck fracture. Color scales are the same for *A*–*F*; *G* and *H* have a slightly expanded scale, with the zero at the same color. Viewpoints are labeled in *A* and are consistent throughout.

Figure 2 shows the result of running the SPM analysis over all subjects (actually the patches used in the statistical models are very slightly different, because each of these leaves one subject out; however, the effect of this variation is almost invisible in this figure). Results are shown for all CBM variables, though only CM and ECTD were subsequently used in calculating hazard ratios and in the predictive models. These results are shown as percentage difference with respect to the mean value at each point. This is slightly problematic for ECTD, because the average trabecular bone mineral density can be very near to zero, and hence a small absolute change may lead to a large percentage change. Nevertheless, we prefer to visualize percentage changes, because they are more closely linked to the statistically significant regions: a larger absolute change is generally required in an area with larger mean value to attain significance.

**Figure 3:**
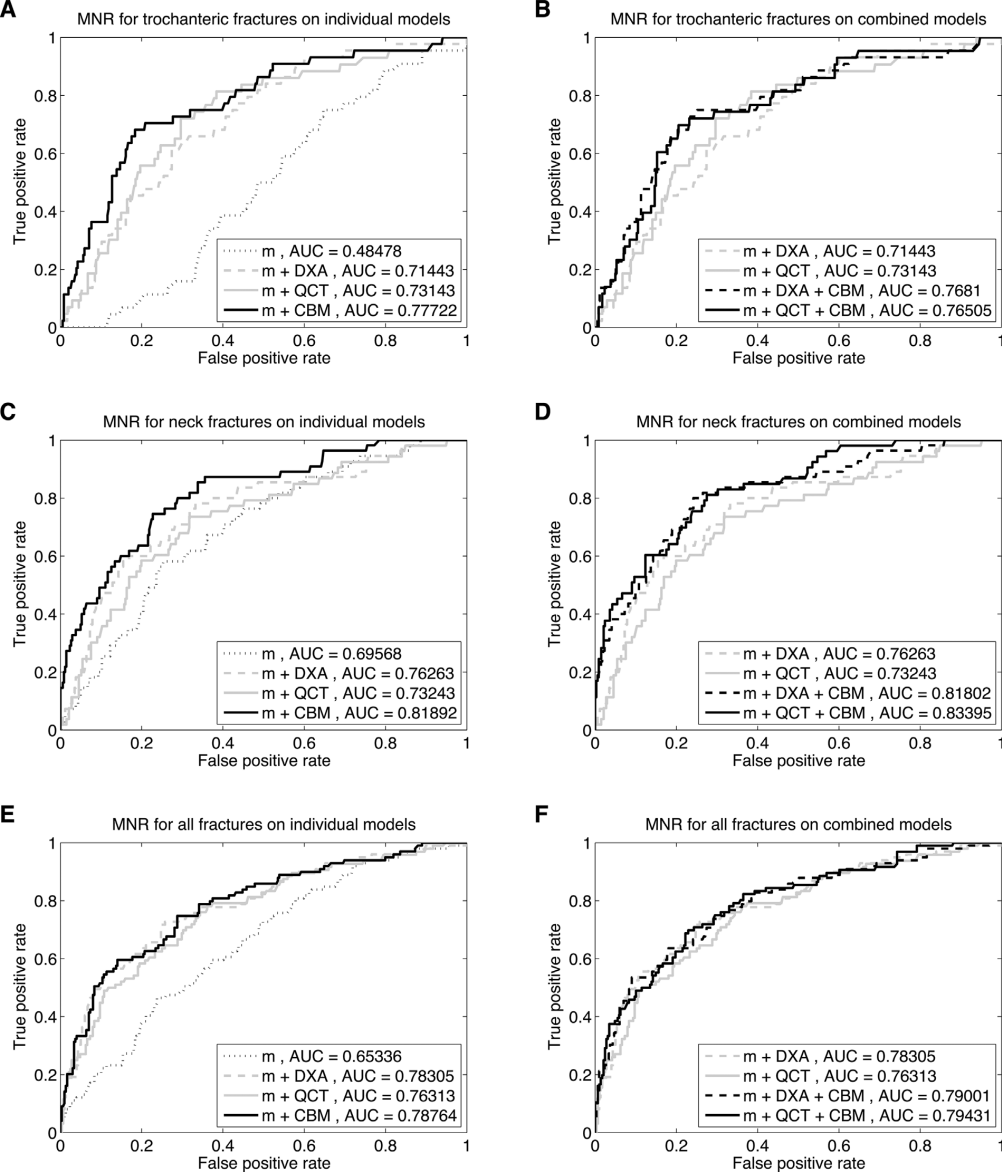
ROC curves for fracture prediction based on leave‐one‐out cross‐validation of MNR models. The base model m includes age + height + site. (*A*, *C*, *E*) The left‐hand graphs show results for either of DXA, QCT, or CBM variables added to this base model. (*B*, *D*, *F*) The right‐hand graphs show results for adding CBM variables to models already containing either DXA or QCT variables. The top row shows trochanteric fracture prediction (*A*, *B*), the middle row neck fracture prediction (*C*, *D*), and the bottom row prediction of any type of fracture (*E*, *F*). Numerical results for these models are given in Table 4. MNR = multinomial logistic regression.

Hazard ratios (from Cox regression) and odds ratios (from binomial and multinomial logistic regression) are shown in Tables 2 and 3, respectively. These contain ratios for fractures of any type, as well as for specific (trochanteric or neck) fracture types. Predictive models were assessed using leave‐one‐out cross validation, and subsequent ROC curves are shown in Fig. 3. Areas under these curves (AUCs) were calculated, with confidence bounds determined by the bootstrap technique, and these are contained in Table 4. The models were assessed for significant difference by comparing the deviances of nested models, using a χ^2^ test. This is a goodness‐of‐fit test that directly compares how well the model fits the data: in this case how closely the fracture probabilities output for each subject from each model (after cross‐validation) match the actual fracture events. It is only valid for comparing nested models; ie, two models where one model is a subset of the other.

**Table 2:** Hazard Ratios for Each 5‐Year Increase (Age), 1 SD Increase (Height), or 1 SD Decrease (All Others) in Quantity

	All fractures		Trochanteric fracture		Neck fracture	
Quantity	Hazard ratio	95% CI	Hazard ratio	95% CI	Hazard ratio	95% CI
Age	1.81^a^	1.47–2.23	1.52^a^	1.15–2.02	2.10^a^	1.62–2.73
Height	0.97	0.79–1.19	0.94	0.71–1.23	0.99	0.76–1.28
DXA ThBMD	2.86^a^	1.98–4.12	3.52^a^	2.24–5.54	2.56^a^	1.63–4.01
DXA FnBMD	3.65^a^	2.30–5.78	3.86^a^	2.07–7.22	3.70^a^	2.05–6.69
QCT ThBMD	3.28^a^	2.11–5.11	3.94^a^	2.22–7.01	2.94^a^	1.70–5.09
QCT FnBMD	2.80^a^	1.83–4.28	2.88^a^	1.71–4.82	2.82^a^	1.63–4.88
CM trochanter patch	2.34^a^	1.67–3.28	3.45^a^	2.13–5.58	1.80^a^	1.24–2.62
CM neck patch	3.00^a^	2.06–4.38	2.80^a^	1.80–4.35	3.32^a^	1.98–5.58
ECTD trochanter patch	3.70^a^	2.39–5.72	4.63^a^	2.59–8.30	3.25^a^	1.86–5.66
ECTD neck patch	4.87^a^	2.91–8.14	4.52^a^	2.51–8.13	5.36^a^	2.57–11.18

Hazard ratios calculated from an unadjusted model (for age and height) or age + height + site + quantity (for all others).

^a^ Significance is given for *p* < 0.005.

**Table 3:** Odds Ratios for 10‐Year Fracture Incidence for Each 5‐Year Increase (Age), 1 SD Increase (Height), or 1 SD Decrease (All Others) in the Quantity

	All fractures		Trochanteric fracture		Neck fracture	
Quantity	Odds ratio	95% CI	Odds ratio	95% CI	Odds ratio	95% CI
Age	1.75^a^	1.40–2.18	1.41	1.05–1.89	2.11^a^	1.58–2.81
Height	1.06	0.83–1.37	1.00	0.71–1.40	1.12	0.82–1.54
DXA ThBMD	2.58^a^	1.91–3.50	2.96^a^	1.98–4.43	2.32^a^	1.62–3.32
DXA FnBMD	3.08^a^	2.19–4.34	3.11^a^	1.99–4.85	3.07^a^	2.02–4.67
QCT ThBMD	2.68^a^	1.94–3.71	3.21^a^	2.07–4.97	2.33^a^	1.59–3.42
QCT FnBMD	2.33^a^	1.69–3.20	2.46^a^	1.61–3.74	2.21^a^	1.50–3.27
CM trochanter patch	2.28^a^	1.70–3.06	3.31^a^	2.15–5.09	1.78^a^	1.27–2.50
CM neck patch	2.52^a^	1.86–3.41	2.40^a^	1.63–3.55	2.61^a^	1.79–3.82
ECTD trochanter patch	3.01^a^	2.17–4.17	3.49^a^	2.23–5.45	2.69^a^	1.83–3.96
ECTD neck patch	3.33^a^	2.35–4.72	3.08^a^	1.98–4.80	3.57^a^	2.31–5.50

Odds ratios calculated from a model of age + height + site (for age and height) or age + height + site + quantity (for all others).

^a^ Significance is given for *p* < 0.005.

**Table 4:** Cross‐Validated AUCs for Various Predictive Models

	Binomial			Multinomial				
	All fractures			Trochanteric fracture		Neck fracture		
Model	AUC	95% CI	DEV	AUC	95% CI	AUC	95% CI	DEV
m	0.653	0.59–0.71	425	0.482	0.40–0.57	0.695	0.62–0.76	562
m + DXA	0.783	0.72–0.83	369^a^	0.714	0.63–0.78	0.762	0.69–0.83	508^a^
m + QCT	0.763	0.70–0.82	365^a^	0.731	0.64–0.80	0.732	0.65–0.80	498^a^
m + CBM	0.787	0.73–0.84	361^a^	0.777	0.68–0.84	0.818	0.75–0.87	466^a^
m + DXA + CBM	0.790	0.73–0.84	362	0.767	0.68–0.84	0.817	0.75–0.87	471^c^
m + QCT + CBM	0.794	0.74–0.85	343^b,c^	0.765	0.67–0.83	0.834	0.77–0.89	446^b,c^
m + all DXA	0.782	0.73–0.83	372^a^	0.699	0.61–0.78	0.777	0.69–0.84	508^a^
m + all QCT	0.756	0.69–0.81	370^a^	0.704	0.62–0.79	0.719	0.63–0.79	513^a^
Combined model 1	0.779	0.72–0.84	359^a^	0.689	0.60–0.76	0.760	0.69–0.82	497^a^
Combined model 2	0.783	0.72–0.83	355^a^	0.700	0.62–0.77	0.757	0.68–0.82	498^a^

AUCs calculated using binomial (all fractures) or multinomial (specific fractures) logistic regression. The base model m includes age + height + site. Significance (based on DEV) is given for *p* < 0.005, based on the model compared to ^a^m, ^b^m + CBM, or ^c^the same model without CBM variables. The last 4 models were selected postanalysis: “all DXA” includes all 4 listed DXA variables; “all QCT” includes all 9 listed QCT variables; “Combined model 1”^28^ is m + DXA FnBMD + QCT trabecular FnBMD; and “Combined model 2”^18^ is m + DXA FnBMD and ThBMD + all 3 QCT trabecular BMD values.

AUC = area under the receiver‐operating characteristic curve; DEV = deviance.

**Figure 4:**
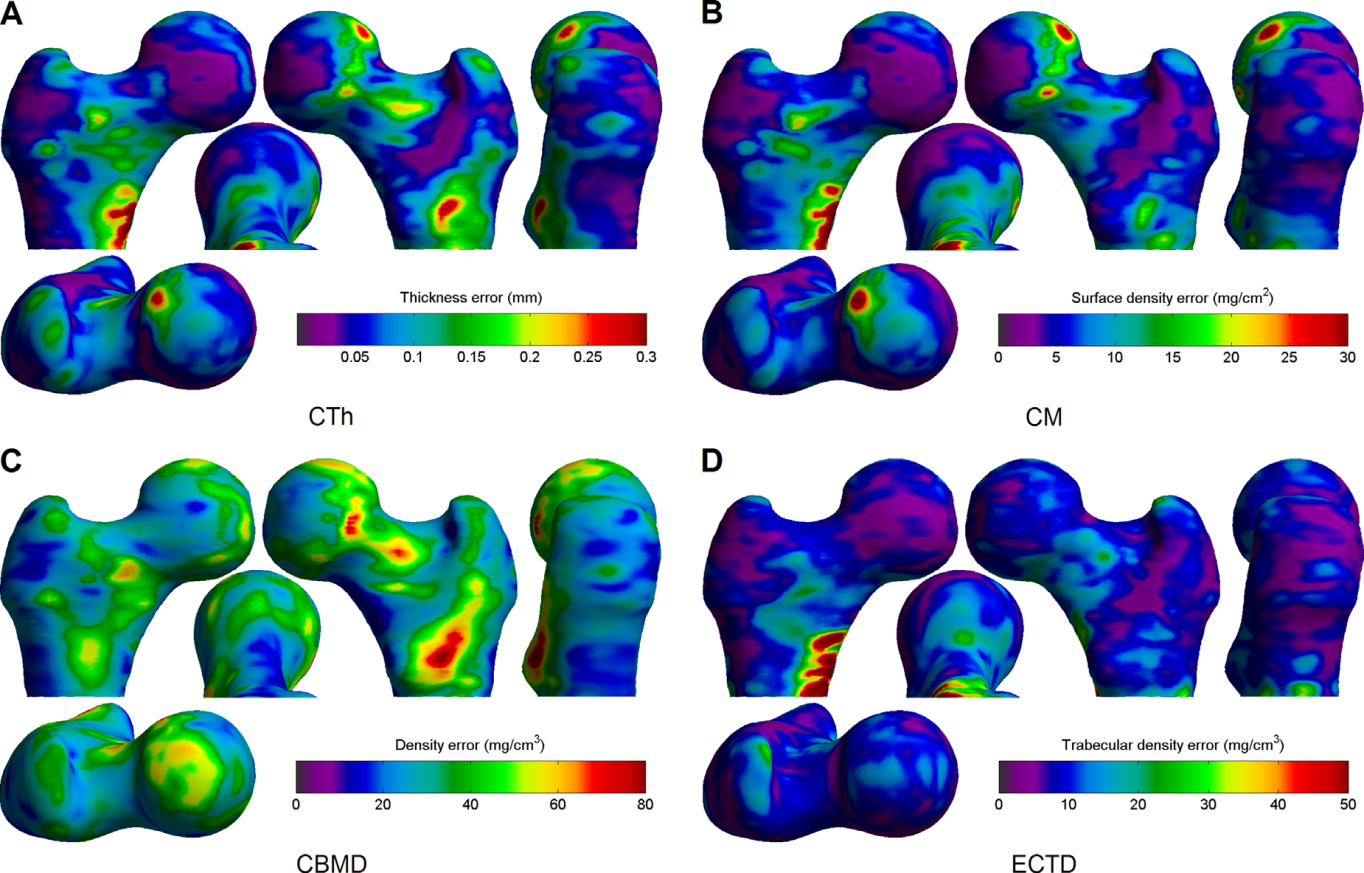
Precision of local CBM measurements. The images show how the absolute measurement error (SD) of each CBM variable varies over the femoral surface: CTh (*A*); CM (*B*); CBMD (*C*); and ECTD (*D*). Viewpoints are the same as described in Fig. 2 *A*.

The precision of CBM variables was also assessed and the estimated measurement SDs are given in Table 5. This is reported as an absolute value, percentage of the mean value (percentage coefficient of variation), and also as a percentage of the SD of that variable in the full cohort. This latter value gives a better indication of how useful this measurement is as a predictor, because it is approximately one‐half the minimum detectable difference for a single subject, at 95% confidence. The distribution of measurement error is also given in Fig. 4.

## Discussion

Looking at the baseline figures in Table 1, the results for DXA‐ and QCT‐based BMD largely confirm what has already been widely reported: that all of these measures are clearly predictive of fracture, though it is interesting to note that this is the case for either class of fractures, irrespective of whether BMD is measured over total hip or femoral neck. Weight, height, and age results agree well with a previous discriminative study on women,^20^ confirming that age is a stronger predictor, as well as discriminator, of fracture than either weight or height. However, age shows much more significance for neck fracture than for trochanteric fracture, which may well be related to the particular regions associated with age‐related bone loss^23^ and how these compare to those regions associated with each fracture type in Fig. 2. In contrast, reduced weight shows significance for trochanteric but not neck fractures, though at a fairly low significance level.

### CBM

The first step in assessing how CBM might contribute to fracture risk is to detect over which parts of the proximal femur each CBM variable is associated with fracture; this is shown in Fig. 2, for CTh, CM, CBMD, and ECTD. The distribution and percentage changes in cortical thickness and cortical mass surface density show a striking similarity to those found by the same method in a published independent discriminative study in women.^11^, ^28^ In both cases, particularly with CM, there is a clear patch at the superolateral side of the trochanter associated with trochanteric fracture, and an even clearer patch at the superior femoral neck associated with neck fracture. This might not be a surprising result in general; in fact it would be worrying if CBM variables were not located in regions associated with that fracture type, but nevertheless it is striking that the shape and location of these patches is so consistent between studies. The CBM patches are also fairly consistent with a previous study using a voxel‐based analysis on a smaller female cohort.^18^ Most of the cases in that study (29/38) sustained neck fractures, and the detected voxel‐based regions associated with fracture are hence most similar to the ECTD neck patches in Fig. 2.

Looking at CBMD distribution in Fig. 2 *E* and *F*, this does appear to be associated with trochanteric fracture, and in regions which are appropriate to that fracture type; however, the percentage difference is much smaller between cases and cohort. When this is considered in light of the substantially poorer precision of this measurement as seen in Table 5 and Fig. 4, then it would seem unlikely that this will perform well as a predictor, even if there are some detectable differences in fracture cases. In addition, the relatively low variation of CBMD between cases and cohort underlies the similarity between the CTh and CM distributions. Hence our prior decision to include neither CTh nor CBMD in the predictive models seems justified: in the former case there is similar information and at slightly better precision in CM, and in the latter case the CBMD changes are much too small compared to the measurement precision.

**Table 5:** Estimated Precision (SD) of Measurement Repeatability

	Precision		
Quantity	Absolute	% of Mean (% coefficient of variation)	% of SD in cohort
CTh (mm)	0.099	6.22	9.8
CTh trochanter patch (mm)	0.033	1.06	2.97
CTh neck patch (mm)	0.027	1.07	2.41
CM (mg/cm^2^)	9.14	5.20	7.27
CM trochanter patch (mg/cm^2^)	3.98	1.32	2.46
CM neck patch (mg/cm^2^)	3.08	1.15	2.20
CBMD (mg/cm^3^)	34.7	3.17	42.3
CBMD trochanter patch (mg/cm^3^)	17.5	1.63	26.6
CBMD neck patch (mg/cm^3^)	19.3	1.76	26.5
ECTD (mg/cm^3^)	15.0	8.81	21.2
ECTD trochanter patch (mg/cm^3^)	2.47	1.49	3.72
ECTD neck patch (mg/cm^3^)	2.63	1.55	4.20

CBM precision is shown both for an individual measurement, and for all measurements aggregated within each of the trochanteric and neck patches. The right‐hand column shows the precision as a percentage of the SD seen in the entire cohort.

The ECTD distribution in Fig. 2 *G* and *H*, on the other hand, shows quite dramatic differences between fracture cases and cohort, and between the fracture types. Although this difference is significant over much of the proximal femur, the distributions are quite distinct between trochanteric and neck fractures. As with CM, CBM patches for trochanteric fracture are focused on the superolateral trochanter, and neck fractures at the superior femoral neck, with substantially less involvement on the inferomedial side. This is a particularly interesting result, given that it is known that the inferomedial femoral neck is largely preserved with age,^6^ and may go some way to explaining why this preservative effect does not prevent neck fracture risk increasing with age. It should be noted here that, although ECTD is clearly very significant, the percentages in Fig. 2*G* and *H* (which have an increased scale compared to the other subfigures) need to be interpreted with care given that the mean ECTD can approach a value close to zero.

We have already discussed the relatively poor performance of the CBMD measurement compared to the other CBM variables. Looking at the other precision values in Table 5 clarifies that this is largely due to the small variation of this variable in the cohort: all point‐wise errors are at around 5% of the mean value, but CBMD has a much smaller cohort variation. The distribution of these errors in Fig 4 reveals that there is a repeatable pattern to where they tend to occur. For CTh and CM, there are larger errors around the femoral head and also at the medial side of the lesser trochanter. The former is due to the presence of the acetabulum, which is very close to the femur at this point and makes it harder to separate out the respective cortices in this region. In the latter area, the cortex is often not well modeled as a single layer and the measurements are less precise as a result. Fortunately, most of the patches in Fig. 2 *A*–*D* are not coincident with these high‐error regions, and hence the CBM precision after aggregation over these patches is substantially better, at about 1% of the mean. ECTD is not affected by the acetabulum but suffers similar imprecision at the medial side of the lower trochanter.

### Fracture prediction

As expected, hazard ratios in Table 2 are significant for all BMD measures, with QCT‐based ThBMD the most significant for trochanteric fracture (and for any fracture), but DXA‐based FnBMD is the quantity of choice for neck fracture. In contrast to QCT, the DXA‐derived areal BMDs contain some measure of size as well as volumetric density, and this may contribute to the improved performance for neck fracture from what is otherwise a less direct measurement of BMD. Nevertheless, hazard ratios between DXA and QCT BMD are largely similar, supporting the general results from previous studies that fail to show any significant benefit from adding such QCT measurement to fracture predictors which already include DXA measurements.^19^, ^20^, ^29^

Hazard ratios for CM are similar to those for BMD; however, ECTD hazard ratios are somewhat larger, a similar result to that found by Yang and colleagues,^19^ where their trabecular volumetric BMD measurements also tended to be the best predictors. This is not a surprising result, because their work was based on data from the same study, but it confirms that the result holds true for our larger sample size, and indicates that our measurement of endocortical trabecular density is capturing the important information contained within the trabecular compartment. All CBM patches show greater hazard ratios for the fracture type on which they were based.

The odds ratios for 10‐year fracture incidence in Table 3 are somewhat smaller than the hazard ratios, because the predictive model to which these lead is designed only to detect whether a subject will fracture, not how long it will be before they fracture. Hence there is some loss of information: it may be presumed that those who fracture a long time after the initial baseline scan had relatively thicker or stronger bones at baseline than those who fractured soon afterward. The predictive model does not take advantage of this information, but nevertheless is necessary in order to evaluate how well we can predict fracture outcome. Although the results here for DXA and QCT BMD are again quite similar, it is the DXA‐derived FnBMD that has the higher odds ratio for all fractures, as well as for neck fractures: QCT‐based ThBMD is still higher for trochanteric fracture. However, as with hazard ratios, ECTD ratios are the most significant.

Of more importance is how well these quantities perform when combined into predictive models. The ROC curves for just age and height in the left hand graphs of Fig. 3 show that any useful fracture prediction that these might give is purely due to neck fractures: these variables have little power to predict trochanteric fracture. Adding DXA‐based BMD greatly improves performance, although it is clear that ability to predict trochanteric fracture is still much more limited than for neck fracture, a similar result to a recent study on the use of finite element analysis in fracture prediction.^30^ AUCs for these models in Table 4 are somewhat smaller than have occasionally been recorded in the literature. Similar models, including DXA variables, have reported AUCs for any fracture type of 0.86^19^ and 0.80,^20^ rather than our value of 0.78. The former was based on the same study as this current work, though with a different cohort and a smaller number of cases. Hence we conclude that our lower result is probably due to the increased number of cases in this study, or the different use of cross‐validation techniques. Neither of these previous publications specify how the AUC results were validated, and it is worth noting that, had we not used leave‐one‐out cross‐validation in this present work, all of our AUC results would have increased by up to 0.03, bringing them more in line with previous results.

In an attempt to compare model performance with other studies, we have also included four further models, with more DXA and QCT variables, and also with combinations that are more similar to good models from previous works. It might be expected that increasing the granularity of these variables would improve the differentiation of fracture type, but from Table 4 this does not seem to be the case: the models with all parameters do not perform any better. The combined models 1 and 2, similar to those from previous works,^19^, ^29^ do seem to perform better than the individual DXA or QCT models on predicting any fracture (though a direct test is not possible since these models are not nested), but they offer no improvement in differentiating between fractures.

Looking at Fig. 3, it can be seen that any of the models which are based on CBM results are more capable of distinguishing between fracture types, with significantly increased AUCs for both neck and trochanteric fracture. It is clear that CBM improves the ability to distinguish fractures in prediction, and this improvement remains significant when adding CBM quantities to models which already have either DXA‐based or QCT‐based BMD values in them. In contrast, adding DXA to a CBM‐based model does not result in a significant improvement, though the combination with CBM and QCT‐based BMD does increase overall fracture prediction slightly.

DXA is currently the reference imaging modality for fracture risk assessment, and it is unlikely that its use will change for the sake of a small improvement in overall fracture risk. However, if a CT scan is available it could easily be reanalyzed for a CBM‐based fracture prediction, and such concurrent screening is already under investigation in some areas.^31^ Of more immediate importance, though, are the CBM patches that arise out of this analysis. These provide a clear treatment target for regimes aiming to reduce risk of fracture. Both exercise and drug regimes improve bone in specific areas, and these are the areas that appear to be most important.

This study has a number of limitations, many of them common to other analyses of the MrOS data and considered in more detail elsewhere.^21^, ^29^ Of particular note in this respect is the inclusion of multicenter QCT data, which resulted in the necessity to model site as a variable: the likelihood is that some inconsistencies were due to differences in CT machines which were not entirely eliminated by calibration. The study was performed in men, most of whom were white, and hence the conclusions are fully supported only for this demographic. In addition, ideally it would have been better to predetermine the CBM patch location from an independent data set rather than from the MrOS data, although including the patch definition within the cross‐validation process went some way to addressing this issue. It has already been noted that the CBM patches are quite similar to those we had already identified in another study involving women^11^, ^28^: this at least hints both that the shape of the patches might not be dramatically different for other demographics, and that the conclusions may hence also be similar in these other scenarios.

In conclusion, CBM results in a small but statistically significant improvement in fracture prediction, even when added to existing DXA‐based or QCT‐based BMD. However, it is considerably better at predicting risk for each type of fracture, as well as providing a clear treatment target for reducing this risk.

## Disclosures

KESP and GMT are inventors on a related patent application WO2011/042738. This does not alter the authors' adherence to all *JBMR* policies on sharing data and materials.
